# From medical school to global health leadership: 35-year career outcomes and gender disparities from the Aga Khan University Medical College

**DOI:** 10.1186/s12909-025-07602-z

**Published:** 2025-07-15

**Authors:** Adil H. Haider, Maham Vaqar, Asma Altaf Hussain Merchant, Sharjeel Ahmad, Komal Abdul Rahim, Namra Qadeer Shaikh, Noreen Afzal, Shayan Shah, Anum Rahim, Saad Bin Zafar Mahmood, Saqib Kamran Bakhshi, Sadaf Khan, Muhammad Tariq

**Affiliations:** 1 Dean and Professor of Surgery, Medical College, Aga Khan University, Karachi, Pakistan; 2 Medical College, Aga Khan University, National Stadium Road, Karachi, 74800 Pakistan; 3 Dean’s Office, Medical College, Aga Khan University, Karachi, Pakistan; 4 Department of Community Health Sciences, Aga Khan University, Karachi, Pakistan; 5 Internal Medicine, Medical College, Aga Khan University, Karachi, Pakistan; 6 Neurosurgery, Medical College, Aga Khan University, Karachi, Pakistan; 7 Associate Dean and Professor of Surgery, Medical College, Aga Khan University, Karachi, Pakistan

**Keywords:** Medical education, Alumni outcomes, Gender disparities, Healthcare leadership, Career trajectories, Alumni survey

## Abstract

**Background:**

Medical education plays significant role in shaping the future of healthcare, and understanding the career outcomes of medical graduates provides valuable insights into the effectiveness of educational programs. With no published data using alumni surveys in the South Asian region, we set out to conduct a survey to gain insights into the career trajectories, professional milestones, and accomplishments of 35 graduating classes from one of Pakistan’s largest Academic Medical Centers (AMC).

**Methods:**

An online survey was distributed to 2,177 alumni of the Aga Khan University (AKU), Pakistan who graduated from 1988 to 2021. This cross-sectional survey explored graduates’ higher education, training, practice settings, and four key outcomes: awards, leadership roles, research impact, and contributions to healthcare and educational innovations. A multipronged approach leveraging outreach from leadership, social media engagement, peer-to-peer appeals, event-based promotion, and targeted text reminders to maximize survey participation was employed. Data were analyzed using descriptive statistics, chi-square tests for association, and multivariable logistic and multinomial regression to assess independent predictors of career outcomes.

**Results:**

1,201 alumni (55.2%) responded to the survey, demonstrating the effectiveness of this multifaceted approach. After the exclusion of missing data, 862 responses were analyzed. Both genders were equally represented (49.8%). Most participants had completed residency (82.0%) and fellowship (56.0%) training. Nearly half (48.5%) were employed in an academic setting. A proportion (57.7%) of alumni had received awards, and nearly one-third of the respondents (31.6%) were involved in developing healthcare or educational innovations. Over half of the respondents (53.7%) had served in leadership roles, and a number had been involved in research (68.9%), with 18.6% receiving grant funding. While an overwhelming 95% of female respondents were employed, they had lower odds of serving in a leadership role and research involvement than their male counterparts.

**Conclusion:**

The findings of this study serve as a testament to the effectiveness of AKU’s educational programs in preparing graduates to make significant contributions to healthcare and society. Continuous quality improvement initiatives, fostering a culture of giving back within its alumni, and creating opportunities for females through diverse endeavors can pave the way for sustained and heightened accomplishments among its graduates.

**Supplementary Information:**

The online version contains supplementary material available at 10.1186/s12909-025-07602-z.

## Introduction

Medical education institutions must continually evolve to ensure the addition of competent physicians to the workforce. Alumni surveys have emerged as powerful tools for assessing an institution’s success in shaping the careers of its graduates [1]. These surveys capture a variety of indicators, including job satisfaction, salaries, professional accomplishments, and civic and political engagement [2, 3]. Moreover, they provide valuable insights into graduates’ satisfaction with their educational experience, highlighting program strengths and areas for improvement [2, 3]. It also enables the quantitative measurement of an institution’s contribution to patient care and science.

With growing emphasis on gender diversity in medical institutions, women have made substantial contributions to the field of medicine. However, despite the use of alumni surveys to evaluate an institution’s graduates’ achievements, a comprehensive comparison between male and female alumni responses has been lacking. In general, female physicians are reported to have lower salaries, slower career advancement, fewer leadership opportunities, and lower career satisfaction compared to their male counterparts [4–8]. However, as these disparities become increasingly apparent, efforts towards gender parity in medicine and beyond are gaining traction globally [9, 10]. Feedback from alumni surveys can inform institutional adaptation designed to overcome potential barriers to empowering women in the medical profession.

In South Asia, persistent gender disparities in medicine are shaped by a complex interplay of cultural and institutional factors. Societal expectations often prioritize family responsibilities for women and reinforce traditional gender roles, which can limit women’s career advancement and participation in the workforce. Institutional barriers, such as limited mentorship, fewer leadership development opportunities, and implicit or explicit gender bias in training and promotion, further exacerbate these disparities. Studies from Pakistan and neighboring countries have documented that female physicians frequently encounter bias in mentorship, operating room opportunities, and career progression, as well as lack of recognition in the workplace [11–13]. These factors collectively contribute to the underrepresentation of women in leadership and research roles, despite near-equal enrollment in medical schools. Addressing these deeply rooted challenges requires both cultural change and targeted institutional interventions.

Several countries in the South Asian region share similar cultural values, including gendered career expectations. Karan et al. found that 25% of female medical graduates in India are not employed in the healthcare sector [13]. Conversely, a study from Bangladesh showed an increase in both the number of female medical students and physicians over a 9-year period [14]. Therefore, it is important to assess whether the notion of female medical graduates not joining the workforce is actually true for all countries in the region and identify how institutions differ in the contributions of their female graduates to the medical workforce.

The Aga Khan University Medical College (AKU-MC) in Pakistan has fostered a rich and diverse alumni network since its establishment in 1983 [15]. AKU is currently ranked among the top 100 universities globally for clinical medicine as per Shanghai Ranking’s Global Ranking of Academic Subject 2019 [15]. Since its inception, AKU has graduated many physicians, making significant contributions to healthcare. Its aim is to prepare individuals for leadership roles, shape public and private policies, and achieve this through research excellence and education. As the university celebrates its 40th anniversary in 2023, this study examines the institution’s evolution and impact by assessing 30 graduating classes of the Bachelor of Medicine and Bachelor of Surgery (MBBS) program, contributing not only to the institution’s understanding of its influence but also to the global discourse on medical education outcomes research. This study also seeks to explore gender differences in career achievements among medical graduates and identify potential disparities, providing insights that can help shape future educational strategies.

## Methods

### Study setting

AKU is located in Karachi, Pakistan, and was the first private university in the country [15]. The medical college offers a 5-year MBBS program that has undergone multiple curricular modifications since inception. Currently, AKU utilizes an integrated spiral curriculum [15], admitting 100 students annually. The program focuses on acquiring early clinical skills during the first two years and employs a problem-based learning (PBL) methodology. As students’ progress through Years 3, 4, and 5, there is a gradual shift towards problem-solving, patient-based learning, and evidence-based practice, enabling students to incorporate the latest research findings and clinical knowledge into their learning. During Year 5, students function as sub-interns with increased patient-care responsibility under the supervision of faculty and residents.

### Study design and participants

This study employed a cross-sectional survey design. Participants included all alumni with valid email addresses who graduated from AKU between 1988 and 2021, spanning over 35 graduating classes. The university has a dedicated Office of Alumni Affairs, which maintains a record of the mailing addresses of all alumni. This list was used to reach out to eligible participants for the survey.

### Study tool and data collection

Based on a comprehensive literature search and the authors’ extensive experience in medical education (MT and AHH), a survey was designed to capture data on alumni career pathways and various indicators of career achievements. In addition to demographic data, the survey collected details about specialized medical education training and other educational milestones such as pursuing advanced degrees (e.g., master’s or PhD). The survey also invited respondents to report their career achievements across four distinct categories: awards received, leadership positions held, contributions to healthcare and educational innovations, and research impact. These categories served as key indicators of alumni accomplishments in their respective fields.

The survey underwent pilot testing with a group of graduates and selected faculty, and revisions were made based on their feedback to ensure clarity and comprehension of the survey items. The final survey was then disseminated via email using the Qualtrics platform over two months, from January 9, 2023, to March 9, 2023. An informed consent form preceded the survey. To provide participants with context regarding the survey’s purpose and use of data, a reference to the publicly available 2016 AKU Alumni Survey Report was included [16]. 

### Survey promotion strategies

At the three-week mark, the survey response rate stood at only 10%. To address this challenge, a multifaceted promotional strategy was implemented based on evidence from the literature on enhancing survey participation among alumni and physicians [17, 18]. Leadership endorsements played a central role in boosting engagement; personalized email reminders from prominent university leaders such as the Dean, Vice Dean, and Associate Dean replacing standard weekly reminders from the alumni office resulted in immediate surges in responses, with 100–200 additional submissions recorded within 24 h. In addition to reminders posted on social media platforms including Facebook groups, Twitter, Instagram, email reminders by AKUAANA (Aga Khan University Alumni Association of North America) leveraged AKU’s strong alumni network in North America to amplify outreach efforts. To further personalize communication, class representatives from each graduating class were identified to share the survey link within their WhatsApp class groups. This approach aimed to leverage peer-to-peer engagement, as messages delivered by classmates were expected to resonate more strongly with alumni compared to institutional communication from the alumni office. Additionally, the survey was promoted at three key events: an MOU signing ceremony between AKU and AKUAANA, a monthly meeting of program directors (many of whom were alumni), and an alumni reunion where a QR code linked to the survey was shared with attendees. These in-person interactions provided an opportunity for direct engagement with alumni leaders and attendees. Finally, text message reminders were sent to approximately 400–500 alumni for whom contact information was available during the last two weeks of data collection. Respondents who confirmed survey completion were encouraged to disseminate the survey link within their networks and post reminders on their respective alumni groups. All strategies were employed concurrently to ensure broad outreach across diverse communication channels and helped boost the final response rate to 55.2%.

### Statistical analysis

Analyses were performed using STATA 14.0 for Windows (Stata Corp. 2015. College Station, TX, USA). Descriptive statistics were computed for all the variables. Categorical variables were presented as frequencies and percentages. Association between two variables was assessed using a chi-square test of independence with a p-value of less than 0.05 considered statistically significant. Multiple logistic regression was used to assess independent factors leading to higher achievement within the four main outcomes (awards received, leadership roles, contribution to healthcare and educational innovations, and research involvement). Multiple multinomial regression revealed independent factors leading to a higher number of publications.

Categorical variables were coded as binary indicators (e.g., gender: male = 1, female = 0), and multi-category variables were coded using reference groups as appropriate. Given the conceptual distinction between our predictors and the limited number of variables included in each model, we did not formally assess multicollinearity. Odds ratios (ORs) and 95% confidence intervals (CIs) were reported for all models.

## Results

From 2,177 eligible alumni, 55.2% (*n* = 1,201) completed and returned the questionnaire. Since graduates of recent years were in the early stages of their postgraduate training and education, our analysis was limited to responses from alumni who graduated from 1988 to 2019, leading to 1,035 records. Of these, 862 provided consent for using their information in research after accounting for incomplete or partially filled responses.

Among the respondents, both genders were equally represented (*n* = 429, 49.8%), with 4 (0.5%) respondents opting not to disclose their gender. Table 1 provides an overview of the demographic and program-related characteristics of the alumni. Most of our alumni resided in the United States of America (70.9%), followed by Pakistan (18.0%).

**Table 1:** Alumni demographic and Program-Related characteristics

Variable	**N = 862**
	**n (%)**
**Gender**	
Male	429 (49.8)
Female	429 (49.8)
Prefer not to disclose	4 (0.5)
**Country of Residence**	
Pakistan	155 (18.0)
United States of America	611 (70.9)
Canada	24 (2.8)
United Kingdom	35 (4.1)
United Arab Emirates	11 (1.3)
Others	26 (3.0)
**Programs completed at AKU**	
MBBS	710 (82.4)
MBBS & Internship	129 (15.0)
MBBS & Residency	3 (0.3)
MBBS & Graduate Degree	4 (0.5)
MBBS, Internship, Residency & Fellowship	2 (0.2)
MBBS, Internship, Residency, Fellowship & Graduate Degree	14 (1.6)
**Residency**	
AKU	9 (1.0)
Outside AKU	690 (80.0)
Both AKU and Outside AKU	8 (0.9)
Residency not completed	155 (18.0)
**Current Employment Status**	
Employed Full-time	594 (68.9)
Employed Part-time	51 (5.9)
Pursuing further training	108 (12.5)
Self-Employed	68 (7.9)
Looking for Employment	23 (2.7)
Retired	1 (0.1)
Others (taking a break, etc.)	17 (2.0)
**Type of Practice**	
Academic	418 (48.5)
Non-Academic	254 (29.5)
Not Specified	190 (22.0)

Most respondents were employed (95.2%), with 418 (48.5%) working in an academic setting. Overall, 95% of female respondents were employed, with a significant majority (61.6%) employed full-time.

### Graduate medical education

Most of the respondents had completed residency training (*n* = 707, 82.0%), with Medicine (47.1%) and Surgery (15.2%) being the most common specialties pursued. A higher percentage of males pursued residency than females in both medicine (53.6% vs. 46.4%) and surgery (71% vs. 29%). Additionally, 36 residents (5.1%) pursued a residency in multiple specialties. A significant proportion of respondents had also completed a fellowship (56.0%). (See Supplement 1 which displays the distribution of medical specialties pursued by graduates).

Several respondents chose to pursue advanced degrees, including a Masters or PhD, with the most common being Master of Public Health (MPH) (*n* = 49, 5.7%). Additionally, 24 respondents (2.8%) obtained a Master of Business Administration (MBA) degree, and 53 (6.1%) respondents reported completing diplomas and other degrees. There were 39 (4.5%) respondents who also completed their graduate or doctorate degrees.

### Career achievements

#### Awards and recognition

A total of 497 (57.7%) respondents reported receiving awards, highlighting their achievements across five distinct categories: teaching, research, clinical service, innovation, and others (humanitarian, congressional etc.). Teaching awards were the most common (*n* = 135, 15.7%), followed by research (*n* = 124, 14.4%) and clinical service (*n* = 115, 13.3%). Males received more awards than females in all categories. Awards were further categorized based on their level of recognition (departmental, institutional, national, and international). Table 2 presents a comprehensive overview of the distribution of awards among male and female alumni and their corresponding levels of recognition.

**Table 2:** Award distribution by gender and level of recognition

Award categories	Number of alumni who received an award	Gender		Level of Receiving Award				
		Males	Females	Departmental Level	Institutional Level	National Level	International Level	More than one level
Teaching	135 (15.7)	85 (19.8)	50 (11.7)	45 (33.3)	48 (35.6)	3 (2.2)	1 (0.7)	38 (28.1)
Research	124 (14.4)	78 (18.2)	46 (10.7)	14 (11.3)	29 (23.4)	30 (24.2)	12 (9.7)	39 (31.5)
Clinical service	115 (13.3)	71 (16.6)	44 (10.3)	39 (33.9)	41 (35.7)	13 (11.3)	1 (0.9)	21 (18.3)
Innovation	36 (4.2)	24 (5.6)	12 (2.8)	2 (5.6)	10 (27.8)	12 (33.3)	5 (13.9)	7 (19.4)
Other	85 (9.9)	43 (10.0)	42 (9.8)	12 (14.1)	22 (25.9)	34 (40.0)	8 (9.4)	9 (10.6)

#### Leadership roles

Over half (53.7%) of the respondents had served in key leadership roles across various categories. Among the leadership roles assumed by alumni, educational leadership was the most common (10.6%), closely followed by clinical service (10.4%) and administrative leadership (7.1%). While females were prominent in educational leadership, males took the lead in clinical service and administrative leadership roles. (See Supplement 2 which shows Leadership areas and Alumni Contribution to Healthcare and Educational Innovations).

Nearly a quarter of the respondents (23.8%) indicated their involvement in educational leadership roles. Most served as program directors (10.2%), with males slightly outnumbering females (52.4% vs. 47.6%). This was followed by the role of medical director (*n* = 10, 4.9%), where females held the majority (60.0% vs. 40.0%).

### Contribution to healthcare and educational innovations

A total of 272 respondents (31.6%) indicated their involvement in developing healthcare or educational innovations. The participants’ contributions spanned various domains (See supplement 2). Among the various educational innovations, curriculum development emerged as the primary focus for respondents (8.1%), with females leading this domain (77.3% vs. 22.7%). Educational pathways was another common domain of innovations (3.7%), with equal contribution from males and females.

### Research impact

**Fig. 1:**
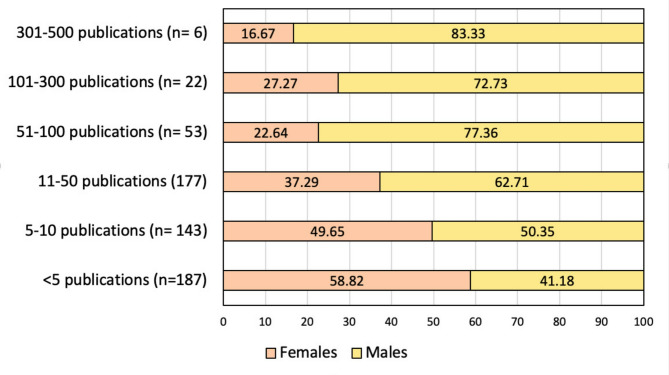
Distribution of Number of Publications Among Alumni

Among the alumni surveyed, a significant proportion (*n* = 591, 68.9%) reported their involvement in research, with 45.0% females (*n* = 266) and 55.0% males (*n* = 325). In addition to this, 160 respondents (18.6%) had received grants, 90 respondents (10.5%) were editorial board members, and 208 (24.2%) respondents served as reviewers for peer-reviewed journals. Males constituted the majority for all categories compared to females, with a marked difference among reviewers (34.7% males vs. 13.8% females). Figure 1 illustrates the distribution of the number of publications among the surveyed alumni, where males had an overall higher number of publications than their female counterparts.

Table 3 shows the adjusted odds ratios with 95% CI for career and achievement outcomes. Males had significantly higher odds of holding a leadership position [aOR 1.54; 95% CI 1.14, 2.08]. Similarly, the odds of being involved in research was 2.21 times more in males compared to females. In addition, males were more likely to receive awards or contribute to healthcare and educational innovations than females.

**Table 3:** Adjusted odds ratios with 95% CI for career and achievement outcomes using multiple logistic regression

Variables	Adjusted Odds Ratio (95% CI)			
	Awards Received	Leadership roles held	Contribution to healthcare and educational innovations	Research Involvement
**Gender** (Ref: Female)				
Male	1.30 (0.98, 1.72)	**1.54 (1.14**,** 2.08)**	1.24 (0.91, 1.69)	**2.21 (1.61**,** 3.05)**
**Years since Graduation**	1.00 (0.98, 1.01)	**0.91 (0.89**,** 0.93)**	**0.94 (0.92**,** 0.96)**	**1.03 (1.01**,** 1.05)**
**Practice Setting** (Ref: non-academic)				
Academic	**1.80 (1.30**,** 2.50)**	**1.93 (1.36**,** 2.75)**	**2.31 (1.61**,** 3.30)**	**4.61 (3.22**,** 6.60)**

**Fig. 2:**
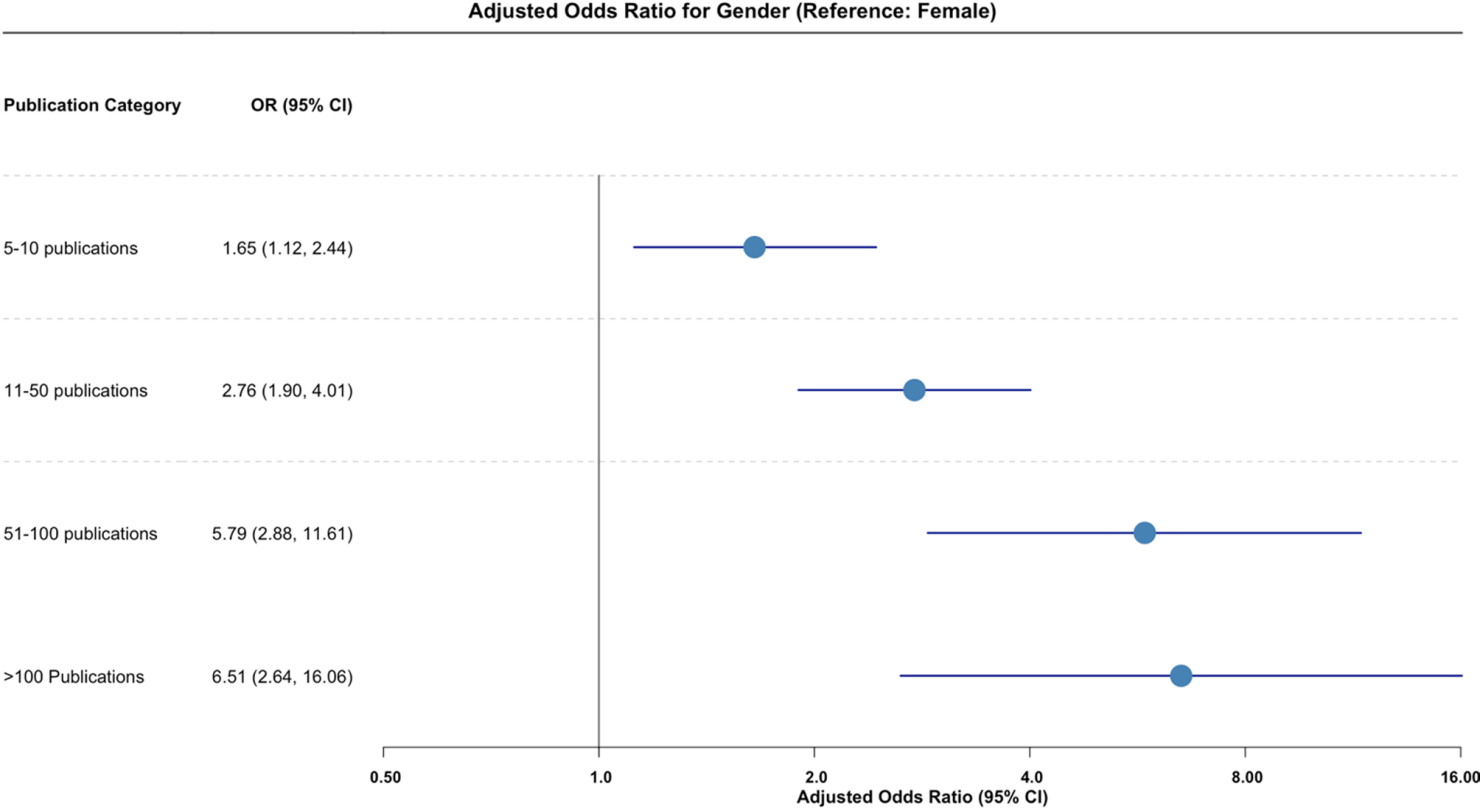
Forest plot illustrating adjusted odds ratios (95% confidence intervals) for publication count, stratified by gender The model was also adjusted for practice setting (academic vs. non-academic)

Figure 2 shows the adjusted odds ratios with 95% CI for the number of publications using multinomial regression. Overall, males had significantly more publications in all categories compared to females, with the highest likelihood of having > 100 publications (AOR 6.51; 95% CI 2.64, 16.06).

## Discussion

Through this large-scale survey, we report unique career outcomes of medical graduates from one of the largest Academic Medical Centers (AMCs) in South Asia. With over half of recipients completing the questionnaire, our findings revealed that most of the alumni were based in the United States, with substantial contribution to healthcare through their leadership, innovations, and research output. The results further provide details of alumni achievements from a gender perspective, which has not been explored in prior alumni surveys from other institutions. While male graduates were more likely to report receiving awards, holding leadership roles, and contributing to research, female graduates reported greater involvement in educational leadership roles.

Achieving a 55.2% response rate in this alumni survey is particularly notable, given the historically low participation rates in physician-focused surveys due to demanding professional commitments [19]. Personalized emails from senior institutional leaders including the Dean and Vice Dean generated immediate surges of 100–200 responses within 24 h, consistent with evidence that personalized outreach can enhance response rates [20]. Social media platforms (Facebook, WhatsApp) and class representatives facilitated peer-to-peer dissemination within established alumni networks, while event-based promotions (e.g., MOU signings, departmental reunions) enabled direct engagement through QR codes and in-person appeals. Final-phase text reminders to 400–500 alumni, coupled with respondent-driven word-of-mouth sharing, further extended reach. While it is challenging to isolate the impact of individual strategies due to their concurrent implementation, our experience suggests that this multipronged approach was effective in achieving broad coverage and maximizing participation across diverse respondent demographics. Different individuals are likely to respond to different modes of outreach; therefore, employing multiple strategies simultaneously ensured that we reached as many potential respondents as possible. Collectively, these adaptive, multichannel strategies - integrating leadership engagement, peer networks, and digital and event-based outreach - demonstrate the value of combining digital, interpersonal, and institutional strategies to boost alumni survey response rates among medical graduates.

More than half of the respondents had completed residency and fellowship training to enhance their expertise in specialized areas, with medicine and its subspecialties being the most common specialty pursued. Notably, around 80.0% of our graduates reported pursuing residency outside AKU, with the majority relocating to the United States of America for further training. In 2021, 22.9% international medical graduates (IMGs) were pursuing their post-graduation as residents and fellows in the United States at ACGME (Accreditation Council for Graduate Medical Education) accredited institutions [21], with Pakistan being within the top five countries of origin of these IMGs [22, 23]. While this trend is often described in terms of “brain drain,” our findings and recent institutional experience suggest a more nuanced reality. Many AKU alumni who train abroad return to Pakistan or remain actively engaged with the local health system, bringing back international standards of practice, innovation, and leadership. The concept of beneficial brain drain suggests that graduates migrating abroad may eventually offer more value to their home countries because of access to better training and the potential to deliver better outcomes [24]. Alumni working abroad can serve as ambassadors, foster international collaborations, and potentially contribute to capacity-building efforts in their home country through knowledge transfer, mentorship, and philanthropic engagement.In our study, more than 50% of graduates reported excelling in further training and practice, as reflected by awards, leadership roles, and research output. A recent example is the Tele-ICU initiative taken during the COVID-19 pandemic [25]. This novel national-level tele-medicine model reported outcomes comparable to international data and was crucial in the face of inadequate critical care resources throughout the country [26]. Studies have also reported the benefits of IMGs on diversifying the workforce in terms of helping inculcate cultural competence in their peers and potentially increasing access to healthcare in rural areas [27–29]. 

Awards and recognition are vital in identifying individuals with exceptional contributions and symbolizing professional achievement [30]. A notable proportion of alumni (41.5%) reported receiving awards for their outstanding achievements in teaching, research, clinical service, innovation, and other areas. In our survey, males consistently reported receiving more awards in all domains than females. This finding is consistent with literature indicating that women in the health workforce receive fewer recognition awards [31, 32]. A 48-year review of award recipients by the American Academy of Physical Medicine and Rehabilitation (AAPM&R) concluded that 84.1% were males compared to 15.9% were females [33], indicating subpar recognition of female health workers globally. Since no other alumni survey focuses on the recognition of their graduates by gender, our results address a gap in the literature and call for attention to the recognition of female healthcare providers within their respective fields. This opportunity holds potential for empowering women physicians, a particularly crucial initiative in low-resource settings like Pakistan, where 50% of female graduates face challenges in pursuing medicine after graduation [34]. 

Studies indicate that effective leadership and management in healthcare positively impact patient outcomes and overall healthcare performance [35]. Over half of our survey respondents (53.7%) reported serving in diverse leadership roles, where educational leadership positions predominantly included program directors, clerkship directors, and curriculum committee chairs. This suggests that AKU graduates may have a significant influence in shaping educational practices and initiatives. Compared to Pakistan’s overall statistics, our graduates tend to attain more leadership positions after their undergraduate MBBS studies [36]. Furthermore, our findings are comparable to international data in which the 2018 MD-PhD alumni survey from Yale School of Medicine reported 60% of their alumni in leadership roles [37], while 14.4% of the Uniformed Services University of the Health Sciences alumni reported their roles in educational leadership [38]. This presence of AKU alumni in leadership roles indicates their capacity to shape healthcare policies, influence organizational decisions, and spearhead positive changes in the healthcare sector. In our study, it was interesting to note that while male graduates had significantly higher odds of being in a leadership role, female graduates surpassed them in educational leadership positions. While females have generally been underrepresented in leadership positions [39, 40], a changing trend has been reported in specific medical fields such as radiology and oncology, where they have equal, if not more, leadership roles in education [41, 42]. AKU has a longstanding commitment to gender equity, exemplified by initiatives such as organizing the 2019 conference on Gender Equity and Women in Leadership and publishing policy recommendations to advance women’s representation in leadership roles [43]. In addition, AKU has recently launched the Women Leadership Academy as the next step towards expanding their leadership skills [44]. Such initiatives during undergraduate training have the potential to increase female representation in healthcare leadership.

A notable proportion of respondents (31.6%) also reported involvement in developing healthcare or educational innovations, with curriculum development being the most common area of contribution. This may reflect the leadership qualities fostered by AKU’s curriculum, which emphasizes high standards in teaching and content expertise [15]. Among healthcare innovations, alumni had contributed the most to the advancement of telemedicine, indicating its recognition as a pivotal area for the future of medicine [45]. This recognition was particularly pronounced during the COVID-19 pandemic [46], which not only heightened the focus on telemedicine but also propelled its rapid progress in response to the unprecedented challenges posed by the pandemic.

Medical advancement relies heavily on scientific research, highlighting the importance of physicians actively bridging the gap between research and patient care by communicating and implementing the latest clinical and translational research findings [47]. The involvement of 68.9% of respondents in research suggests a strong culture of evidence-based practice among the alumni. Similar findings were noted in the UMass Chan Medical School alumni survey, where 54% of research occurred at the Academic Health Science Center [48]. The successful acquisition of grant funding for research endeavors further demonstrates the impact and productivity of AKU graduates in research. Many alumni had also served as editorial board members or reviewers for peer-reviewed journals, contributing to the dissemination of scientific knowledge. From a gender perspective, our results indicate that males reported significantly higher research representation and output than their female counterparts. Multiple studies have reported similar findings, with male physicians in academic positions publishing more research overall [49–51]. Our findings highlight the need to create more research opportunities for females, both for publications and grants, and to promote gender equity in medical education and practice.

The Thomas Jefferson University’s longitudinal study, initiated in 1970, is the longest and most comprehensive endeavor tracking medical graduates from a single Academic Medical Center (AMC) [52]. Spanning the past five decades, it has followed its medical students and graduates retrospectively and prospectively. The exhaustive data gathered in this database has been instrumental in evaluating the university’s curriculum and gauging its long-term efficacy [53]. Similar endeavors include the Pennsylvania model, whereby six medical schools collaborated on a state level to track their students from enrollment to later careers [54]. Our study contributes to the literature on medical education and alumni outcomes by providing novel findings, as published data using similar alumni surveys have not been reported from South Asia; thus, our study offers unique insights from this part of the world. Our response rate of 55.2% is more than double that of the 2019 Survey at UMass Chan Medical School in Worcester, Massachusetts (26%) [48], and also exceeded rates from other alumni surveys in the United States and the United Kingdom [55, 56]. Furthermore, previous alumni surveys rarely report findings by gender, and our study helps bridge this gap by critically evaluating alumni performance and opportunities available after graduation by gender.

Limitations of this study include potential response bias and the reliance on self-reported data. While efforts were made to reach a diverse sample of AKU graduates, respondents who chose to participate in the survey may represent a subset of individuals with higher levels of achievement or involvement. Additionally, self-reported data may be subject to recall or social desirability biases [57]. Our study follows graduates from only one medical school in South Asia and the findings may not be generalizable to other institutions or countries in the region, given differences in educational environments, resources, and cultural contexts. Future research in collaboration with other regional medical schools would allow for comparative analyses and a more comprehensive understanding of alumni outcomes and gender disparities across South Asia. While our study highlights gender disparities, a more in-depth exploration of the root causes behind these disparities is warranted. Future research endeavors could incorporate qualitative methods or longitudinal tracking to better understand the factors influencing career trajectories and achievements.

Looking ahead, we recognize the limitations of cross-sectional alumni surveys in capturing the full scope of career trajectories and the impact of educational interventions. In light of this, our institution is currently exploring the feasibility of establishing a comprehensive longitudinal database of medical education outcomes, inspired by successful initiatives such as the Jefferson Longitudinal Study of Medical Education [52]. Such a database would enable systematic tracking of students and graduates from admission through their professional careers, facilitating robust longitudinal analyses of factors influencing career development, leadership attainment, and gender equity over time. The present study was conceived as a foundational step in this direction, providing baseline data and highlighting key areas for ongoing monitoring and potential future intervention.

## Conclusion

This study is a comprehensive assessment of the accomplishments and influence of medical graduates from a research-intensive institution. It adds to the existing literature on medical education and alumni outcomes. The results strongly support the efficacy of AKU’s educational programs, showcasing the substantial contributions made by graduates to healthcare and society. Moreover, it emphasizes the need to address gender disparities in career achievements and actively create equal opportunities for both males and females in medicine. The insights garnered from this research can guide educational institutions, policymakers, and healthcare organizations in developing future initiatives and policies aimed at enhancing medical education and encouraging the sustained participation of undergraduates in the field of medicine.

## Electronic supplementary material

Below is the link to the electronic supplementary material.

Supplementary Material 1

Supplementary Material 2

## Data Availability

The data for this study were obtained exclusively from the alumni survey conducted by the Aga Khan University. No outside data sources were utilized. The data is available in the tables and figures provided.

## Abbreviations

AKU: Aga Khan University
AMC: Academic Medical Center
MBBS: Bachelor of Medicine, Bachelor of Surgery
MPH: Master of Public Health
MBA: Master of Business Administration
ACGME: Accreditation Council for Graduate Medical Education
IMG: International Medical Graduate
AKUAANA: Aga Khan University Alumni Association of North America
PBL: Problem-Based Learning
CI: Confidence Interval
aOR: Adjusted Odds Ratio
MOU: Memorandum of Understanding
ERC: Ethical Review Committee
MD: Doctor of Medicine
MHPE: Master of Health Professions Education
MPhil: Master of Philosophy
BScN: Bachelor of Science in Nursing
PhD: Doctor of Philosophy
AKU-MC: Aga Khan University Medical College
COVID-19: Coronavirus Disease 2019
Tele-ICU: Tele-Intensive Care Unit
AKUAANA: Aga Khan University Alumni Association of North America
QR: Quick Response
IMGs: International Medical Graduates

## References

[1] Eesley CE. Alumni surveys as a data collection methodology. SSRN Electron J. 2018.

[2] Cabrera AF, Weerts DJ, Zulick BJ. Making an impact with alumni surveys. New Dir Institutional Res. 2005;2005(126):5–17.

[3] Volkwein JF. Assessing alumni outcomes. New Dir Institutional Res. 2010;2010(S1):125–39.

[4] Lo Sasso AT, Richards MR, Chou CF, Gerber SE. The $16,819 pay gap for newly trained physicians: the unexplained trend of men earning more than women. Health Aff (Millwood). 2011;30(2):193–201. 21289339 10.1377/hlthaff.2010.0597

[5] Jagsi R, Biga C, Poppas A, Rodgers GP, Walsh MN, White PJ, et al. Work activities and compensation of male and female cardiologists. J Am Coll Cardiol. 2016;67(5):529–41. 26560679 10.1016/j.jacc.2015.10.038 PMC4962867

[6] Jagsi R, Griffith KA, Stewart A, Sambuco D, DeCastro R, Ubel PA. Gender differences in the salaries of physician researchers. JAMA. 2012;307(22):2410–7. 22692173 10.1001/jama.2012.6183

[7] Weaver AC, Wetterneck TB, Whelan CT, Hinami K. A matter of priorities? Exploring the persistent gender pay gap in hospital medicine. J Hosp Med. 2015;10(8):486–90. 26122400 10.1002/jhm.2400

[8] Olson EM, Kennedy CC, Kelm DJ. Assessment of gender parity: leadership representation in pulmonary and critical care medicine. J Womens Health. 2022;31(3):439–46. 10.1089/jwh.2020.8982 PMC9022127 33956512

[9] Mehta S, Burns KEA, Machado FR, Fox-Robichaud AE, Cook DJ, Calfee CS, et al. Gender parity in critical care medicine. Am J Respir Crit Care Med. 2017;196(4):425–9. 28240961 10.1164/rccm.201701-0076CP PMC6995357

[10] Raj A, Kumra T, Darmstadt GL, Freund KM. Achieving gender and social equality: more than gender parity is needed. Acad Med. 2019;94(11):1658–64. 31335818 10.1097/ACM.0000000000002877

[11] Janjua MB, Inam H, Martins RS, Zahid N, Sattar AK, Khan SM, et al. Gender discrimination against female surgeons: A cross-sectional study in a lower-middle-income country. Annals Med Surg. 2020;57:157–62. 10.1016/j.amsu.2020.07.033 PMC7394833 32774847

[12] Ghimire A, Sharma Neupane M. The hidden curriculum: examining gender disparities in career trajectories of female medical graduates from Nepal. BMC Public Health. 2025;25(1):1555. 40287720 10.1186/s12889-025-22700-9 PMC12032813

[13] Karan A, Negandhi H, Nair R, Sharma A, Tiwari R, Zodpey S. Size, composition and distribution of human resource for health in india: new estimates using National sample survey and registry data. BMJ Open. 2019;9(4):e025979. 10.1136/bmjopen-2018-025979 PMC6549895 31133622

[14] Hossain P, Gupta R, Das, YarZar P, Jalloh MS, Tasnim N, Afrin A, et al. Feminization’ of physician workforce in bangladesh, underlying factors and implications for health system: insights from a mixed-methods study. PLoS ONE. 2019;14(1):e0210820. 30633775 10.1371/journal.pone.0210820 PMC6329528

[15] Vaqar M, Tariq M, Khan MR, Khan S, Riaz Q, Mahmood S, et al. A journey of innovation: 40 years of pioneering medical education at the Aga Khan university medical college in karachi, Pakistan. Postgrad Med J. 2024;100(1183):350–7. 38648192 10.1093/postmj/qgad139

[16] Aga Khan University Alumni Affairs. Alumni Survey Report: 2016. 2017.

[17] Smith K, Bers T. Improving alumni survey response rates: an experiment and cost-benefit analysis. Res High Educ. 1987;27(3):218–25.

[18] Mwizerwa J, Robb W, Namukwaya C, Namuguzi M, Brownie S. Improving response rates to an alumni survey in East Africa. Adv Social Sci Res J. 2017;4(20):120–7.

[19] Cunningham CT, Quan H, Hemmelgarn B, Noseworthy T, Beck CA, Dixon E, et al. Exploring physician specialist response rates to web-based surveys. BMC Med Res Methodol. 2015;15(1):32. 25888346 10.1186/s12874-015-0016-z PMC4404667

[20] Cook C, Heath F, Thompson RL. A Meta-Analysis of response rates in Web- or Internet-Based surveys. Educ Psychol Meas. 2000;60(6):821–36.

[21] 2022 Physician Specialty Data Report Executive Summary| AAMC [Internet]. [cited 2023 Dec 30]. Available from: https://www.aamc.org/data-reports/data/2022-physician-specialty-data-report-executive-summary

[22] How IMGs have changed the face of American medicine| American Medical Association [Internet]. [cited 2023 Dec 30]. Available from: https://www.ama-assn.org/education/international-medical-education/how-imgs-have-changed-face-american-medicine

[23] Ahmed AA, Hwang WT, Thomas CR, Deville C. International medical graduates in the US physician workforce and graduate medical education: current and historical trends. J Grad Med Educ. 2018;10(2):214. 29686763 10.4300/JGME-D-17-00580.1 PMC5901803

[24] Kuhn PJ, McAusland C. The International Migration of Knowledge Workers: When is Brain Drain Beneficial? 2006.

[25] Latif A, hussain SA, Atiq H, Zaki M, Khan H, Sami K, et al. 166: CLINICAL OUTCOMES OF CRITICALLY ILL COVID-19 PATIENTS SEEN THROUGH TELE-ICU SERVICES IN PAKISTAN. Crit Care Med. 2022;50(1):67–67.

[26] Khan MA, Shahbaz H, Noorali AA, Ehsan AN, Zaki M, Asghar F et al. Disparities in adult critical care resources across pakistan: findings from a National survey and assessment using a novel scoring system. Crit Care. 2022;26(1). 10.1186/s13054-022-04046-5 PMC9272593 35818054

[27] Norcini JJ, Van Zanten M, Boulet JR. The contribution of international medical graduates to diversity in the U.S. Physician workforce: graduate medical education. J Health Care Poor Underserved. 2008;19(2):493–9. 18469420 10.1353/hpu.0.0015

[28] Cohen JJ. The consequences of premature abandonment of affirmative action in medical school admissions. JAMA. 2003;289(9):1143–9. 12622585 10.1001/jama.289.9.1143

[29] Hart LG, Skillman SM, Fordyce M, Thompson M, Hagopian A, Konrad TR. International medical graduate physicians in the united states: changes since 1981. https://doi org/101377/hlthaff2641159. 2017;26(4):1159–69. 10.1377/hlthaff.26.4.1159 17630460

[30] Lincoln AE, Pincus S, Koster JB, Leboy PS. The matilda effect in science: awards and prizes in the US, 1990s and 2000s. Soc Stud Sci. 2012;42(2):307–20. 22849001 10.1177/0306312711435830

[31] Silver JK, Blauwet CA, Bhatnagar S, Slocum CS, Tenforde AS, Schneider JC, et al. Women physicians are underrepresented in recognition awards from the association of academic physiatrists. Am J Phys Med Rehabil. 2018;97(1):34–40. 28678034 10.1097/PHM.0000000000000792 PMC5757674

[32] Morgan R, Dhatt R, Muraya K, Buse K, George AS. Recognition matters: only one in ten awards given to women. Lancet. 2017;389(10088):2469. 10.1016/S0140-6736(17)31592-1 PMC6995358 28656894

[33] Silver JK, Bhatnagar S, Blauwet CA, Zafonte RD, Mazwi NL, Slocum CS, et al. Female physicians are underrepresented in recognition awards from the American academy of physical medicine and rehabilitation. PM R. 2017;9(10):976–84. 28336430 10.1016/j.pmrj.2017.02.016

[34] Moazam F, Shekhani S. Why women go to medical college but fail to practise medicine: perspectives from the Islamic Republic of Pakistan. Med Educ. 2018;52(7):705–15. 29508422 10.1111/medu.13545

[35] Sfantou DF, Laliotis A, Patelarou AE, Sifaki-Pistolla D, Matalliotakis M, Patelarou E. Importance of leadership style towards quality of care measures in healthcare settings: A systematic review. Healthcare. 2017;5(4). 10.3390/healthcare5040073 PMC5746707 29036901

[36] Khokhar AJ. Women academic leaders in higher education in pakistan: perspectives of female students enrolled in higher education degrees. Pakistan J Women’s Studies: Alam-e-Niswan. 2018;25(2):59–76.

[37] Yale School of Medicine. 2018 MD-PhD Alumni Survey.

[38] Jung E, McBee E, Schreiber-Gregory DN, Teng Y, Dong T, Durning SJ. Career accomplishments of uniformed services university of the health sciences medical graduates: classes 1980–2017. Mil Med. 2023;188 (Supplement2): 111–4. 37201486 10.1093/milmed/usac235

[39] McKimm J, Da Silva AS, Edwards S, Greenhill J, Taylor C. Women and leadership in medicine and medical education. Int Perspect. 2015;2:69–98.

[40] Carr PL, Raj A, Kaplan SE, Terrin N, Breeze JL, Freund KM. Gender differences in academic medicine: retention, rank, and leadership comparisons from the National faculty survey. Acad Med. 2018;93(11):1694. 29384751 10.1097/ACM.0000000000002146 PMC6066448

[41] Webb EM, Kallianos KG, Vella M, Straus CM, Bucknor MD, Galvan J, et al. Are women disproportionately represented in education compared to other roles in academic radiology?? Acad Radiol. 2020;27(12):1767–73. 32111467 10.1016/j.acra.2020.01.036

[42] Riaz I, Bin, Siddiqi R, Zahid U, Durani U, Fatima K, Sipra Q, ul AR, et al. Gender differences in faculty rank and leadership positions among hematologists and oncologists in the united States. JCO Oncol Pract. 2020;16(6):e507–16. 32048924 10.1200/OP.19.00255

[43] https://www.aku.edu/news/Documents/gender-equity.pdf. The time is now, Gender equity and Women in leadership conference.

[44] The Aga Khan University News. Aga Khan University launches Women Leadership Academy.

[45] Jin MX, Kim SY, Miller LJ, Behari G, Correa R. Telemedicine: current impact on the future. Cureus. 2020;12(8). 10.7759/cureus.9891 PMC7502422 32968557

[46] The Aga Khan university. #AKUHeroes| Alumni.

[47] Salgueira A, Costa P, Gonçalves M, Magalhães E, Costa MJ. Individual characteristics and students engagement in scientific research: A cross-sectional study. BMC Med Educ. 2012;12(1):1–9. 23066758 10.1186/1472-6920-12-95 PMC3515434

[48] UMass. Chan Medical School. Alumni Survey: 2019 Report.

[49] Hart KL, Perlis RH. Trends in proportion of women as authors of medical journal articles, 2008–2018. JAMA Intern Med. 2019;179(9):1285–7. 31135815 10.1001/jamainternmed.2019.0907 PMC6547141

[50] Jagsi R, Guancial EA, Worobey CC, Henault LE, Chang Y, Starr R, et al. The gender gap in authorship of academic medical literature–a 35-year perspective. N Engl J Med. 2006;355(3):281–7. 16855268 10.1056/NEJMsa053910

[51] Fridner A, Norell A, Åkesson G, Gustafsson Sendén M, Tevik Løvseth L, Schenck-Gustafsson K. Possible reasons why female physicians publish fewer scientific articles than male physicians - A cross-sectional study career choice, professional education and development. BMC Med Educ. 2015;15(1):1–8. 25889674 10.1186/s12909-015-0347-9 PMC4404646

[52] Gonnella JS, Hojat M, Veloski J. AM last page. The Jefferson longitudinal study of medical education. Acad Med. 2011;86(3):404. 21346438 10.1097/ACM.0b013e31820bb3e7

[53] Gonnella JS, Erdmann JB, Hojat M. An empirical study of the predictive validity of number grades in medical school using 3 decades of longitudinal data: implications for a grading system. Med Educ. 2004;38(4):425–34. 15025644 10.1111/j.1365-2923.2004.01774.x

[54] Rabinowitz HK, Veloski JJ, Aber RC, Adler S, Ferretti SM, Kelliher GJ et al. A statewide system to track medical students’ careers: the Pennsylvania model. Acad Med. 1999;74(1 Suppl). 10.1097/00001888-199901001-00042 9934320

[55] Seattle UW. UW Alumni Survey Results 2019–2020 DOCTORAL/PROFESSIONAL Degree Recipients. UW Seattle All Professional, School Of Medicine School Of Medicine UW Alumni Survey Results. 2021.

[56] Maisonneuve JJ, Lambert TW, Goldacre MJ. Doctors’ views about training and future careers expressed one year after graduation by UK-trained doctors: questionnaire surveys undertaken in 2009 and 2010. BMC Med Educ. 2014;14(1):1–9. 25528260 10.1186/s12909-014-0270-5 PMC4302441

[57] Kreuter F, Presser S, Tourangeau R. Social desirability Bias in CATI, IVR, and web surveys: the effects of mode and question sensitivity. Public Opin Q. 2008;72(5):847–65.

[58] World Medical Association Declaration of Helsinki. JAMA. 2013;310(20):2191. 24141714 10.1001/jama.2013.281053

