# Correction: From medical school to global health leadership: 35-year career outcomes and gender disparities from the Aga Khan University Medical College

**DOI:** 10.1186/s12909-025-07776-6

**Published:** 2025-08-14

**Authors:** Adil H. Haider, Maham Vaqar, Asma Altaf Hussain Merchant, Sharjeel Ahmad, Komal Abdul Rahim, Namra Qadeer Shaikh, Noreen Afzal, Shayan Shah, Anum Rahim, Saad Bin Zafar Mahmood, Saqib Kamran Bakhshi, Sadaf Khan, Muhammad Tariq

**Affiliations:** 1 Dean and Professor of Surgery, Medical College, Aga Khan University, Karachi, Pakistan; 2 Medical College, Aga Khan University, National Stadium Road, Karachi, 74800 Pakistan; 3 Dean’s Office, Medical College, Aga Khan University, Karachi, Pakistan; 4 Department of Community Health Sciences, Aga Khan University, Karachi, Pakistan; 5 Internal Medicine, Medical College, Aga Khan University, Karachi, Pakistan; 6 Neurosurgery, Medical College, Aga Khan University, Karachi, Pakistan; 7 Associate Dean and Professor of Surgery, Medical College, Aga Khan University, Karachi, Pakistan

**Correction: BMC Med Educ 25, 1054 (2025)**

**https://doi.org/10.1186/s12909-025-07602-z**

Following publication of the original article [1], the authors identified an error in the author name of Muhammad Tariq.

The incorrect author name is Muhammed Tariq.

The correct author name is Muhammad Tariq.

The author group has been updated above and the original article [1] has been corrected.
