# Supplementary material for: From medical school to global health leadership: 35-year career outcomes and gender disparities from the Aga Khan University Medical College
Source: BMC Med Educ. 2025 Jul 15;25:1054. doi: 10.1186/s12909-025-07602-z (PMC12261767; doi:10.1186/s12909-025-07602-z)
Supplement: Supplementary file 1 — Supplementary Material 1 [file 12909_2025_7602_MOESM1_ESM.docx]

**Supplement 1:** Distribution of Medical Specialties among Graduates (n= 705)*

| **Specialty** | **Total**  **n (%)** | **Female**  **n (%)** | **Male**  **n (%)** |
| --- | --- | --- | --- |
| Medicine, specialties & subspecialties | 332 (47.1) | 154 (46.4) | 178 (53.6) |
| Surgery, specialties and subspecialties | 107 (15.2) | 31 (29.0) | 76 (71.0) |
| Pediatrics | 86 (12.2) | 69 (80.2) | 17 (19.8) |
| Family Medicine | 24 (3.4) | 19 (79.2) | 5 (20.8) |
| Emergency Medicine | 6 (0.9) | 2 (33.3) | 4 (66.7) |
| Anesthesiology | 15 (2.1) | 8 (53.3) | 7 (46.7) |
| Pathology | 11 (1.6) | 7 (63.6) | 4 (36.4) |
| Radiology | 15 (2.1) | 7 (46.7) | 8 (53.3) |
| OB/GYN | 5 (0.7) | 3 (60.0) | 2 (40.0) |
| Psychiatry | 17 (2.4) | 12 (70.6) | 5 (29.4) |
| Ophthalmology | 24 (3.4) | 12 (50.0) | 12 (50.0) |
| Other | 27 (3.8) | 11 (40.7) | 16 (59.3) |
| Multiple | 36 (5.1) | 14 (38.9) | 22 (61.1) |
| *While 707 respondents pursued residency, 2 chose not to disclose their gender. Their subspecialties included Medicine, specialties & subspecialties and Radiology. | | | |
