# Supplementary material for: From medical school to global health leadership: 35-year career outcomes and gender disparities from the Aga Khan University Medical College
Source: BMC Med Educ. 2025 Jul 15;25:1054. doi: 10.1186/s12909-025-07602-z (PMC12261767; doi:10.1186/s12909-025-07602-z)
Supplement: Supplementary file 2 — Supplementary Material 2 [file 12909_2025_7602_MOESM2_ESM.docx]

**Supplement 2:** Leadership areas and Alumni Contribution to Healthcare and Educational Innovations

|  | **Total**  **N (%)** | **Females**  **n(%)** | **Males**  **n(%)** |
| --- | --- | --- | --- |
| **Key leadership areas** | **463 (53.7)** | **203 (43.8)** | **260 (56.2)** |
| Educational leadership | 49 (10.6) | 36 (73.5) | 13 (26.5) |
| Research leadership | 12 (2.6) | 6 (50.0) | 6 (50.0) |
| Clinical Service leadership | 48 (10.4) | 13 (27.1) | 35 (72.9) |
| Administrative leadership | 33 (7.1) | 14 (42.4) | 19 (57.6) |
| Leadership in a non-profit organization | 9 (1.9) | 4 (44.4) | 5 (55.6) |
| Leadership role in a local/community organization | 9 (1.9) | 4 (44.4) | 5 (55.6) |
| Leadership role in a corporate/business enterprise | 5 (1.1) | 3 (60.0) | 2 (40.0) |
| Other | 18 (3.9) | 14 (77.8) | 4 (22.2) |
| Multiple | 280 (60.5) | 109 (38.9) | 171 (61.1) |
|  |  |  |  |
| **Educational leadership roles** | **205 (23.8)** | **107 (52.2)** | **98 (47.8)** |
| Program Director | 21 (10.2) | 10 (47.6) | 11 (52.4) |
| Program Coordinator | 5 (2.4) | 4 (80.0) | 1 (20.0) |
| Clerkship Director | 6 (2.9) | 4 (66.7) | 2 (33.3) |
| Section Head | 3 (1.5) | 2 (66.7) | 1 (33.3) |
| Department Chair | 3 (1.5) | 1 (33.3) | 2 (66.7) |
| Curriculum Committee Chair | 1 (0.5) | 1 (100.0) | 0 (0.0) |
| Medical Director | 10 (4.9) | 6 (60.0) | 4 (40.0) |
| Chief Medical Officer | 2 (1.0) | 2 (100.0) | 0 (0.0) |
| Other | 59 (28.8) | 37 (62.7) | 22 (37.3) |
| Multiple | 95 (46.3) | 40 (42.1) | 55 (57.9) |
|  |  |  |  |
| **Healthcare/Educational Innovations** | **272 (31.6)** | **121 (44.5)** | **151 (55.5)** |
| Personalized Medicine | 3 (1.1) | 0 (0.0) | 3 (100.0) |
| Digital health | 4 (1.5) | 0 (0.0) | 4 (100.0) |
| Artificial Intelligence | 0 (0.0) | 0 (0.0) | 0 (0.0) |
| Telemedicine | 4 (1.5) | 3 (75.0) | 1 (25.0) |
| Web-based applications/mobile applications | 2 (0.7) | 2 (100.0) | 0 (0.0) |
| Medical devices | 7 (2.6) | 0 (0.0) | 7 (100.0) |
| Point-of-care diagnostics | 3 (1.1) | 1 (33.3) | 2 (66.7) |
| Educational pathways/tracks | 10 (3.7) | 5 (50.0) | 5 (50.0) |
| Development of a new educational program | 7 (2.6) | 4 (57.1) | 3 (42.9) |
| Educational policy development | 1 (0.4) | 1 (100.0) | 0 (0.0) |
| Curriculum development | 22 (8.1) | 17 (77.3) | 5 (22.7) |
| Other | 39 (14.3) | 17 (43.6) | 22 (56.4) |
| Multiple | 170 (62.5) | 71 (41.8) | 99 (58.2) |
